# Identification of Novel Chemical Entities for Adenosine Receptor Type 2A Using Molecular Modeling Approaches

**DOI:** 10.3390/molecules25051245

**Published:** 2020-03-10

**Authors:** Kelton L. B. dos Santos, Jorddy N. Cruz, Luciane B. Silva, Ryan S. Ramos, Moysés F. A. Neto, Cleison C. Lobato, Sirlene S. B. Ota, Franco H. A. Leite, Rosivaldo S. Borges, Carlos H. T. P. da Silva, Joaquín M. Campos, Cleydson B. R. Santos

**Affiliations:** 1 Laboratory of Modeling and Computational Chemistry, Department of Biological and Health Sciences, Federal University of Amapá, 68902-280 Macapá-AP, Brazil; keltonbelem@hotmail.com (K.L.B.d.S.); jorddynevescruz@gmail.com (J.N.C.); luciaanebarros@hotmail.com (L.B.S.); ryanquimico@hotmail.com (R.S.R.); cleyson.cl@gmail.com (C.C.L.); lqfmed@gmail.com (R.S.B.); 2 Nucleus of Studies and Selection of Bioactive Molecules, Institute of Health Sciences, Federal University of Pará, 66075-110 Belém-PA, Brazil; sayuriota@gmail.com; 3 Laboratory of Molecular Modeling, State University of Feira de Santana, Feira de Santana, 44036-900 Bahia, Brazil; moysesfagundes@gmail.com (M.F.A.N.); fhpharm@gmail.com (F.H.A.L.); 4 Computational Laboratory of Pharmaceutical Chemistry, University of São Paulo, 14040-903 Ribeirão Preto-SP, Brazil; tomich@fcfrp.usp.br; 5 Department of Chemistry, Faculty of Philosophy, Sciences and Letters of Ribeirão Preto, University of São Paulo, 14090-901 Ribeirão Preto-SP, Brazil; 6 Department of Pharmaceutical and Organic Chemistry, Faculty of Pharmacy, Institute of Biosanitary, Research ibs. Granada, University of Granada, 18071 Granada, Spain; jmcampos@ugr.es

**Keywords:** adenosine A_2A_ receptor, virtual screening, molecular insight

## Abstract

Adenosine Receptor Type 2A (A_2A_AR) plays a role in important processes, such as anti-inflammatory ones. In this way, the present work aimed to search for compounds by pharmacophore-based virtual screening. The pharmacokinetic/toxicological profiles of the compounds, as well as a robust QSAR, predicted the binding modes via molecular docking. Finally, we used molecular dynamics to investigate the stability of interactions from ligand-A_2A_AR. For the search for A_2A_AR agonists, the UK-432097 and a set of 20 compounds available in the BindingDB database were studied. These compounds were used to generate pharmacophore models. Molecular properties were used for construction of the QSAR model by multiple linear regression for the prediction of biological activity. The best pharmacophore model was used by searching for commercial compounds in databases and the resulting compounds from the pharmacophore-based virtual screening were applied to the QSAR. Two compounds had promising activity due to their satisfactory pharmacokinetic/toxicological profiles and predictions via QSAR (Diverset 10002403 pEC_50_ = 7.54407; ZINC04257548 pEC_50_ = 7.38310). Moreover, they had satisfactory docking and molecular dynamics results compared to those obtained for Regadenoson (Lexiscan^®^), used as the positive control. These compounds can be used in biological assays (in vitro and in vivo) in order to confirm the potential activity agonist to A_2A_AR.

## 1. Introduction

Rational drug design involves different areas of knowledge, especially those related to biological sciences and health, as well as electronic and steric levels of drug, physicochemical, and hydrophobic parameters related to biological activity [1]. The discovery and introduction of new medicines on the market by a pharmaceutical industry is considered a complex, multifactorial, competitive, and time-consuming activity which needs high investment, involving the application of modern techniques and methodologies [2]. From the design of a project to the introduction of a single drug in the market, there is an average cost of 12 to 15 years in the Design, Development, and Innovation (DD&I) process, which can exceed US$1.2 billion. In an attempt to maximize the time-production relationship, the pharmaceutical industries have developed the combinatorial synthesis strategy, making hundreds or thousands of compounds possible per day, by parallel synthesis or in the form of mixing [3].

The therapeutic effect of a compound, physicochemical parameters, as well as their pharmacokinetic and toxicological properties can be evaluated in silico, in vitro, and/or in vivo. This evaluation saves time and financial resources in the DD&I process, since it eliminates candidates with inadequate properties before proceeding to clinical trials; the failure rate of this process is estimated to be over 90%. The main reasons include loss of clinical efficacy, inadequate pharmacokinetic properties, toxicity, adverse reactions, commercial reasons, and pharmacotechnical limitations [4,5]. 

The digital information is growing exponentially, by about nine times in just five years. Over the past decade, there has been an increase in the amount of available compound activity and biomedical data, owing to the emergence of new experimental techniques [6]. Virtual screening has been widely applied in the early phase of drug discovery, being able to accelerate hit discovery and reduce drug development costs. Thus, computer-aided drug design has a very high reward combined with advances in automated experimentation and sophisticated planning software [7,8]. Many studies have been done using computer-aided drug design, such as Structure-Based Drug Design (SBDD), Ligand-Based Drug Design (LBDD), Pharmacophore-Based Drug Design (PBDD), and Fragment-Based Drug Design (FBDD). Among the different types of drug design methods, PBDD has the highest reliability [9]. A search for potential enzyme inhibitors for new drug development or therapeutic drug repurposing has been carried out with the aid of these methods, such as the study by Wu et al. [10] and Yang et al. [11], who identified mitoxantrone 1 as a new ATP-competitive inhibitor of NEDD8-activating enzyme (NAE) by virtual screening and an inhibitor of the VHL–HIF1α interaction via structure based virtual screening, respectively. 

Adenosine is a naturally occurring metabolite that is ubiquitously distributed throughout the body. It has been shown to accumulate in the extracellular space at the site of inflammation, in response to metabolic stress and cellular damage, and there is evidence that it may play a key role in the preservation of homeostasis [12]. Adenosine A_2A_ receptor (A_2A_AR) protects the body against damage from various diseases, such as asthma, inflammation, pneumonia, atherosclerosis, stroke, heart attack, diabetes-induced kidney disease, and many other diseases. A great clinical interest has thus been generated for agonists development of such receptor and would be potential drugs against these damages, especially in the anti-inflammatory process [13]. A_2A_AR is an important regulator of different microglial functions, such as the release of inflammatory mediators and proliferation. It is involved in cancer, cardioprotection, and specifically a great deal of interest has been focused on the A_2A_ receptor and its role in protective bone recovery and anti-inflammatory activity as well [14,15]. The availability of the crystallographic structure of A_2A_AR gives us an opportunity to place these earlier findings in an atomic 3D context and to use the knowledge of atomic interactions to predict novel ligands or similar compounds that have agonist behavior with better selectivity and therapeutic efficacy—in this case, we have the agonist named UK-432097 ((6-(2,2-diphenylethylamino)-9-((2R,3R,4S,5S)-5-(ethylcarbamoyl)-3,4-dihydroxytetrahydrofuran-2-yl)-*N*-(2-(3-(1-pyridin-2-yl)piperidin-4-yl) ureido)ethyl)-9H-purine-2-carboxamide)), of greater potential, has shown leak efficacy in the clinical tests [16,17]. The agonist UK-432097 [18] was developed by Pfizer and discovered for A_2A_AR-selective agonists for the treatment of inflammation and chronic obstructive pulmonary disease. The safety and efficacy of UK-432097 in chronic obstructive pulmonary disease was terminated as of 17 March 2009—see (http://www.clinicaltrials.gov). However, the compound showed carcinogenic activity and, thus, unsatisfactory results in Phase II clinical trials [13,19]. The prediction of pharmacokinetic, toxicological, and stereo-electronic features by in silico studies would be an interesting way for choosing drugs with inhibitory potential, such as demonstrated in previous studies [17,20,21]. 

**Figure 1:**
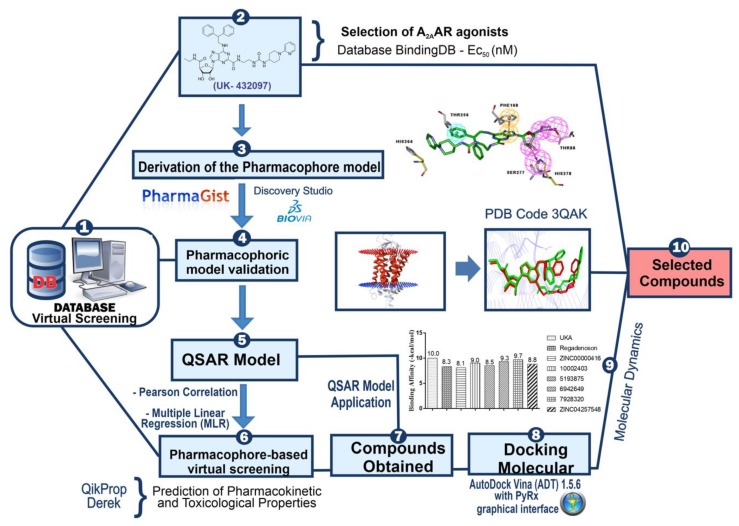
General scheme summarizing of the methodological steps.

In this way, the present work aims to search for compounds via pharmacophore-based virtual screening procedures. In addition, we characterize the pharmacokinetic/toxicological profiles of the compounds, and build a robust quantitative structure–activity relationship (QSAR) model, predicting the binding modes via molecular docking studies, as well. Finally, we use molecular dynamics to investigate the interaction over time in ligand-A_2A_AR. Molecular design and conception of bioactive compounds from virtual screening are studied here—we have as an objective in the search for new chemical entities or scaffolds of potential anti-inflammatory drugs to act on the type 2 adenosine receptor as agonists using molecular modeling approaches. The general scheme summarizing the methodological steps in this paper is presented in Figure 1 (see more details in the materials and methods section).

## 2. Results and Discussions

### 2.1. Pharmacophore Perception Using the PharmaGist and Discovery Studio Softwares 

**Figure 2:**
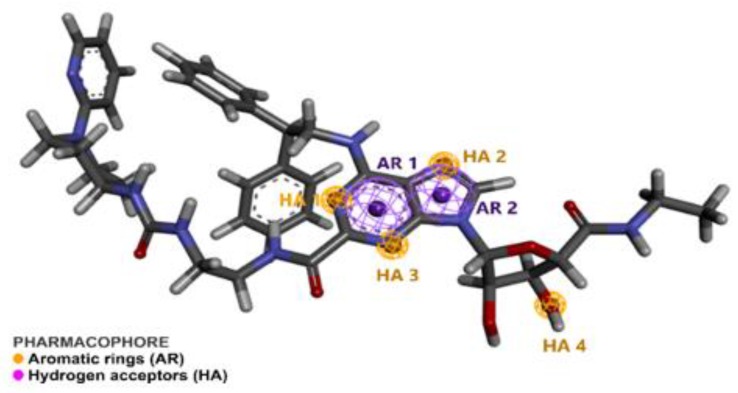
Pharmacophore model of adenosine A_2A_ receptor (A_2A_AR) agonists obtained using the PharmaGist web server. Aromatic rings (AR) are represented in purple and hydrogen acceptor (HA) groups are represented in orange.

Pharmacophore perception for the compounds was carried out using PharmaGist, and the top-ranked pre-alignment of the compounds had a score of 64.605 (see Figure 2), wherein Silva [22] and Padilha [23] independently reported similar score values in their studies, using the same the PharmaGist algorithm. The aligned compounds shared six spatial characteristics: two aromatic (AR) groups and four hydrogen acceptor (HA) groups, where the coordinates and the radius of the spatial characteristics are shown in Table 1. This approach was based on the receptor-ligand complex, it was convenient to locate the binding site of the ligand inside the biological target and to determine the key points of interactions between ligand and target [24,25]; see more details in the materials and methods, Section 3.1. Pharmacophore Detection by Pharmagist and Discovery Studio 4.0.

Pharmacophore hypothese was classified according to the number of ligands aligned or that combined in order to generate a pharmacophore model that was satisfactory based on score, according to literature data [22,23,26]. The pharmacophore model chosen in pre-alignment via PharmaGist was used as input in the Discovery Studio, in order to refine the initial pharmacophore model.

### 2.2. Pharmacophore Model Evaluation

**Figure 3:**
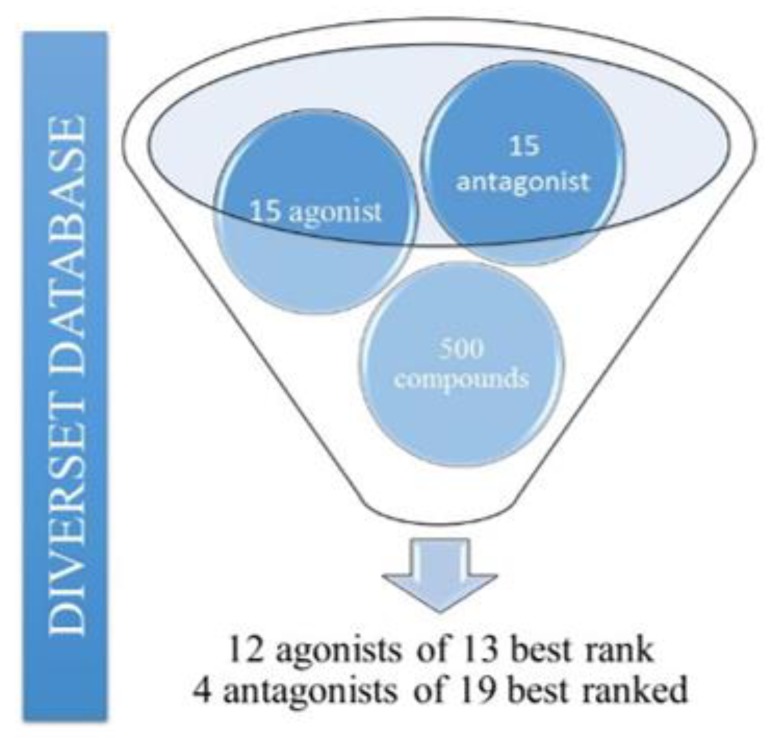
Evaluation of the pharmacophore model selected.

For the pharmacophore model evaluation, 10 models were generated using the Discovery Studio 4.0 software, carried out through the “contamination” of a small part of a subcollection/database, in which 500 unknown random compounds from the Diverset database were selected, along with 30 reference compounds—15 known agonists and 15 antagonists—totalizing a new database with 530 compounds (pharmacophore perception, the most active compounds were considered in order to evaluate molecule groups that can interact with the receptor, as well as the most relevant properties for pharmacophore-based virtual screenings with better efficiency). After the evaluation, the best model selected was the one that recovered 80% of the reference agonist compounds, i.e., 12 agonist compounds among the 13 best-ranked, which means out of a total of 530 compounds the model was able to search 12 agonist-only compounds). Among the 19 best ones (a total of 530 compounds), 4 antagonists were also recovered, see Figure 3. The other models were unsatisfactory in the recovery of the known bioactive ligands, since many hypotheses did not obtain the necessary characteristics for interaction at the A_2A_AR receptor site [26].

**Figure 4:**
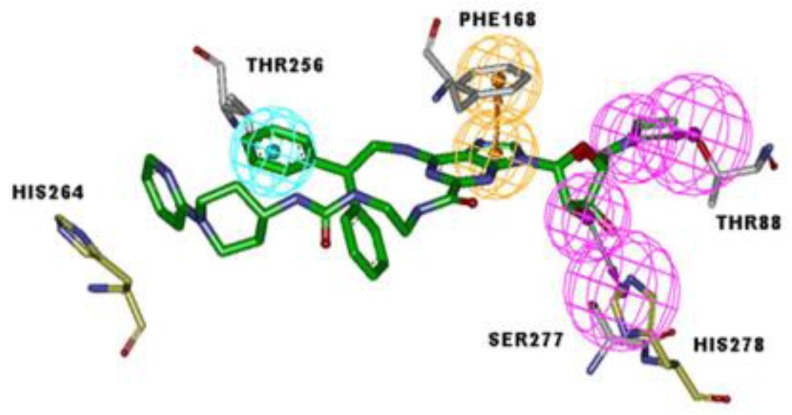
Pharmacophore model selected for the design of novel and potential A_2A_AR agonists, obtained using the Discovery Studio software. In cyan is the sphere representing the hydrophobic group, in rose is the Hydrogen bonding donor and, in orange, the aromatic group.

The pharmacophore chosen from the 10 proposed pharmacophore hypotheses showed features of the ligands which are important to bind the receptor, so that the aromatic ring interacts with the residue PHE168, which plays an important role at the receptor binding site (hydrophobic and π-π-cation interactions). Hydrogen bond acceptor groups of the ribose interact with the residues SER277 and HIS278 of the receptor, and the moiety containing the amide group (-C_3_H_6_ON) interacts with the residue THR88. The pharmacophore model could be used to identify the structurally diverse compounds that have higher probabilities in exhibiting agonist activity and selectivity to A_2A_AR. The pharmacophore hypothesis thus selected, as well as the analysis of the active site, showed that the features are located in regions of interactions with six important residues for biological activity regarding A_2A_AR, as follows: HIS264, THR256, PHE168, HIS278, SER277, and THR88, according the findings from Xu et al. [18]—see Figure 4. 

We observed that the results obtained were congruent, and thus, the proposed pharmacophore model was validated and can be used in future ligand-based virtual screening experiments in order to search for new agonists with potential anti-inflammatory activity, for example. 

The best model selected at this step was used for ligand-based virtual screening (via pharmacophore) in the databases ChemBrigde_DIVERSet, ChemBrigde_DIVERSet_Exp, ZINC_Drug Database, ZINC_Natural_Stock, and ZINC_FDA_BindingDB, in the search for new potential A_2A_AR agonists with better pharmacokinetic, toxicological, and biological activity properties that fit all the features/groups of the pharmacophore model selected here.

### 2.3. QSAR Modeling Using Multiple Linear Regressions (MLR)

After geometry optimization of the most active compounds, their physicochemical and pharmacophore properties were calculated (see Table 2) as well as the Pearson correlation. Descriptors with correlation of 0.50 were used to build the parametric models and six descriptors were selected: Molar Volume (MV), Molecular Polarizability (MP), Number of Atoms (NA), Pharmacophore Features (PF), Hydrophobic Group (HG), and Aromatic (AR)—four pharmacophore descriptors and two physicochemical descriptors with a better correlation with pEC_50_ (pEC_50_ = −logEC_50_). So, a series of 22 parametric equations (15 tetraparametric models, *p* = 4; 6 pentaparametric models, *p* = 5; and 1 hexaparametric model) were obtained through different combinations (no repetitions) using six parameters from the properties indicated by the Pearson correlation. The selected descriptors were used to build the QSAR models, using Equation (1) shown below, based on previous studies [27,28]:

(1) $$Cp,n=\frac{n!}{p!\left(n-p\right)!}$$

where *C* = number of combinations, *p* = model type (*p* ≠ 0 and *p* = 6), and *n* = number of variables (*n* = 6). The QSAR model was built with *n* samples of 16 structures (1, 6, 7, 9–21), since 5 structures were outliers (2–5 and 8—randomic errors contaminated the observations) and they were identified and subsequently removed in order to obtain models with greater predictive power, without impairing the statistical quality that was evaluated by the correlation coefficient (r), squared correlation coefficient (r^2^), explained variance (r^2^_A_, i.e., r^2^ adjusted), standard error of estimate (SEE), and variance ratio (F).

The best regression models were selected based on the higher values of r and F and smaller values of SEE [23]. This was proven through the application of the models to the compounds for verifying the variation between the experimental and theoretical values, calculated through the quantitative structure–activity relationship (QSAR) model.

### 2.4. Tetraparametric Models

The combination of four different parameters was performed so that the combinations did not correlate; thus, 15 tetraparametric regression equations were obtained from the properties selected by Pearson correlation, and the best equation is shown below (Equation (2)):

pEC_50_ = 4.022064 + 0.019606.(MV) − 0.720766.(MP) + 0.216313.(NA) − 0.139588.(HG) (2)

n = 16; r = 0.9457; r^2^ = 0.8944; r^2^_A_ = 0.8560; SEE = 0.3228; F = 23.2970.

This tetravariate combination of molar volume (VM), molar polarizability (PM), number of atoms (NA), and hydrophobic group (GH) showed relevant statistics (r = 0.9457) and explained up to 85.60% of the variance of r^2^_A_. The SEE value was 0.3228 and the F value was 23.2970.

### 2.5. Pentaparametric Models

A series of six pentaparametric equations were obtained through different combinations of five parameters that were not related to each other from the properties selected by Pearson correlation. According to the statistical quality (regression parameters), the best model constructed is shown in Equation (3), below:

pEC_50_ = 4.478449 + 0.023246.(MV) − 0.829411.(MP) + 0.242268.(NA) − 0.081025.(PF) − 0.072964.(HG) (3)

n = 16; r = 0.9634; r^2^ = 0.9281; r^2^_A_ = 0.8922; SEE = 0.279; F = 25.850.

According to Belaid [29], the F value is found to be statistically significant at 95.00% level, since the entire value of F calculated is higher when compared to tabulated values. By Costa [30], a higher F value implies a more significant correlation was achieved. This combination of molar volume (MV), molar polarizability (MP), number of atoms (NA), pharmacophore features (PF), and hydrophobic group (HG) showed relevant statistics (r = 0.9634) and explained up to 89.22% of the variance of r^2^_A_. The SEE value was less than the defined in methods (0.279), while F value was 25.850.

### 2.6. Hexaparametric Model

A combination of six parameters that did not have correlations with each other generated only one hexaparametric regression model, from the properties selected by Pearson correlation. Regression parameters as well as statistical quality are described in Equation (4), below:

pEC_50_ = 3.916999 + 0.025762.(MV) − 0.869548.(MP) + 0.221891.(NA) − 0.081625.(PF) − 0.069166.(HG) + 0.120464.(AR)(4)

n = 16; r = 0.9653; r^2^ = 0.9318; r^2^_A_ = 0.8863; SEE = 0.2868; F = 20.5008.

QSAR models were applied to the set of agonists used for the construction of the models, and the results are found in Table 3. Table 3 shows the applications of QSAR models selected with highest statistical significance, together with theoretical and experimental values, as well as their respective validation errors (residual), calculated by the difference between the experimental and theoretical values, based on studies carried out by Costa et al. [31].

According to the best QSAR models selected, for the tetraparametric model only the compounds **1**, **6**, **9**, **11**–**16**, and **18** showed a lower value of validation error (±0.001180 to ±0.289332), and other compounds used in the model showed a variation ±0.025717 to ±0.391775, where the outliers (compounds **2**–**5** and **8**) had high values, ranging from ±0.574965 to ±2.255648. For the pentaparametric model only compounds **1**, **9**–**12**, **15**, **16**, and **18**-**21** showed a lower value of validation error (±0.002003 to ±0.220591), and other compounds used in the model showed a variation of ±0.327327 to ±0.391775, where the outliers (compounds **2**–**5** and **8**) had high values, ranging from ±0.730975 to ±2.296371.

For the hexaparametric model only compounds **9**–**13**, **15**, **16**, **19**, and **21** showed a lower value of validation error (±0.009363 to ±0.250122), and other compounds used in the model showed variation of ±0.409221 to ±0.800802, where the outliers (compounds **2**–**5** and **8**) had high values, ranging from ±1.736217 to ± 2.719959.

The hydrophobic group (HG) is a characteristic that plays an important role in various chemical, physical, and biological processes. It determines the stability of biological membranes, globular proteins, micelles, and governs the distribution of compounds in living organisms. Although the hydrophobic effect is a well-known and widely-studied phenomenon it is still not well explained. According to Southall [32], hydrophobic groups are features present in molecules with large hydrocarbon groups, that are hydro (water)-phobic (fear), that is, that have no affinity for water, where hydrophobic interaction becomes essential in stability and folding/conformation of protein.

The variation range of the hydrophobic feature was from 1 to 18, where compounds **7** and **20**–**21** had the lowest and highest values, respectively. Compound UK-432097 showed a total of three hydrophobic features. The importance of this descriptor is notorious, since it had the highest Pearson correlation value. This implies that likely hydrophobic interactions could be made by the agonist with the receptor besides hydrogen bond or aromatic interaction (π-π) with nearby amino acids of A_2A_AR, such as THR256. According to the pharmacophore model built here, the action of such a descriptor was observed in compounds **2** and **5**, with values of 0.85 nM and 2.02 nM, respectively. The only one difference between them was an additional methyl (-CH_3_) radical present in the ribose ring of compound **2**, increasing its hydrophobic character, and consequently its biological activity, thus facilitating the interactions with SER277, HIS278, and THR88, according to Figure 4.

**Figure 5:**
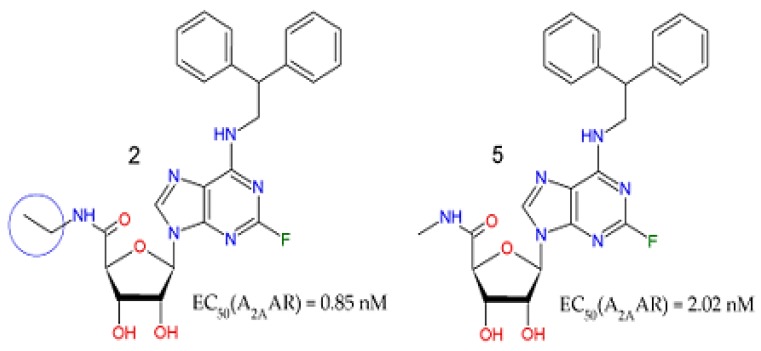
Importance of the hydrophobic feature to A_2A_AR agonism, through the methyl effect.

According to Barreiro et al. [33], the insertion of one or more methyl groups in a bioactive molecule makes it more lipophilic and theoretically less soluble in water. However, in some cases, the insertion of methyl groups into a molecule leads to increased solubility through mechanisms such as reduced intramolecular H-interactions, increased hydrophobic interactions, changes in functional group ionization state, and lowering of the lattice (see Figure 5).

A pharmacophore feature is defined by a set of atoms in the same rigid group with a physicochemical property important for binding, i.e., pharmacophore feature (I–II) is a hydrogen bonding donor-accepting atom; (III–IV) an anion-cation atom; (V) a set of atoms of an aromatic ring (aromatic feature); or (VI) a pair of adjacent hydrophobic atoms [34]. According to Schneidman-Duhovny [35], a set of physicochemical “features” is assigned to the ligands, hydrogen bonding “donor”/“acceptor”, “anion”/“cations”, “aromatic ring”, “hydrophobic group”. It was noted that the descriptor “Pharmacophore Features” (PF) was the sum of the other physicochemical properties/features given by the following Equation (5), observing studies by Dror [34] and Schneidman-Duhovny [35]:

PF = HBD + HBA + CATION + ANION + AR + HG.(5)

The range of variation of the pharmacophore characteristic was 16 to 41, where compounds **7** and **21** had the lowest and highest values, respectively. Compound UK-432097 showed 26 pharmacophore features, see Table 2.

The number of atoms (NA) means the number of atoms present in the structure. The range of the number of atoms varied from 38 to 120, where compounds **7** and **20** showed the lowest and highest values, respectively. Compound UK-432097 had 104 atoms in its structure. Most of the compounds (13 structures) had hydrogen (H), carbon (C), nitrogen (N), and oxygen (O) atoms in addition to these atoms. Compounds **2**, **5**, and **7** had a fluorine atom (F). Compounds 4 and 15 had a bromine (Br) atom, whereas compounds **9** and **18** had a disulfur bridge, i.e., two sulfur atoms connected (R-S-S-R). Only structure 19 had chlorine (Cl) in its chemical composition. 

Molar polarizability (MP) was estimated from the scheme of activity given by Miller [36], with 3% of precision for the calculation, where different variables are associated with different types of atoms; polarization of a molecule characterizes the ability of its electronic system to be distorted by the external field, which plays an important role in molecular modeling regarding many biological properties and activities. A strong van der Waals interaction is a good measure of high polarizability. Highly polarizable molecules may have strong attractions with other molecules, since they may also increase aqueous solubility [29].

In dipole interaction models, the magnitude of the induced dipole moment (μp) in a P atom is proportional to its atomic polarizability (αp), because it plays a fundamental role in calculations of polarization. Polarizability is an essential parameter in the development of a polarizable field of force. The atomic polarizability parameters were obtained through appropriate experimental measurements of molecular polarizabilities or quantum mechanical or quantum mechanical or electrostatic potentials [37]. 

The range of molar polarizability (MP) calculated here was 29.04 to 94.42 A^3^, and compounds **7** and **20** showed the lowest and highest values, respectively. This shows that compound **20** is more susceptible to distortion by an electronic field, thereby increasing its solubility in water. However, in compound **7**, because its area is smaller, it suffers less influence from the electronic field, being less soluble in water.

Observing the value obtained and described on the Pearson correlation, there was an inversely proportional relationship between polarizability and biological activity, i.e., the lower the polarizability (distortion in the electronic field) the greater its biological activity, with the activity value of compound **7** being much greater than compound **20**. Compound UK-432097 had a surface and polarizable area of 82.39 Å^2^, but was more active. This stemmed from the interactions that occur in some specific amino acids of the receptor, such as HIS264 and THR256. The aromatic feature (AR) results from the presence of aromatic rings in its structure. It is important to observe the interactions of compound UK-432097 with A_2A_AR (PDB ID 3QAK), where it interacts aromatically with the receptor. According to Rodríguez [38], an aromatic interaction π-π stacking is observed between the adenine ring of the ligand with PHE168 of the receptor.

The essential chemical features/properties required by an agonist to activate A_2A_AR were shared by most of the agonists: the aromatic nature of the nucleobases for π-π stacking with PHE168 and the stabilizing interactions with LEU249 and ILE274 of the receptor. These molecular features of nucleoside agonists are crucial for recognition of the ligand by the receptor site, where they are considered essential for molecules with biological activity [39]. 

The range of variation of the aromatic feature obtained here was 3 to 6, and the compounds with the lowest values were 3, 4, 7, 9-11, and 15, whereas the compounds with the highest values calculated were 20 and 21. 

The molecular volume (MV) determines the transport characteristics of molecules, responsible for intestinal absorption or blood–brain barrier penetration, for example. Volume is therefore widely used in QSAR studies to model molecular properties and biological activity. In Table 1, the range of molar volume calculated here was 846.19 to 2393.81 A^3^, and compounds **7** and **20** showed the lowest and highest values, respectively. Compound UK-432097 had a molar volume of 2155.82 A^3^.

### 2.7. Quantitative Structure–Activity Relationship (QSAR) Modeling—External Validation

After evaluating the parameters and the statistical quality, the parametric models (tetra-, penta- and hexaparametric) were applied to the set of seven compounds (22-28) in this study, called the test set (totaling 43.75% in relation to the number of compounds used in the training set; *n* = 16). Compounds were selected from the Pubchem database based on their respective EC_50_ values, which were converted to pEC_50_. The molecular properties were calculated; only those used in QSAR models constructed and extracted similarly from the training set. Table 4 shows the selected properties of the test set compounds with their respective biological activity values.

Table 5 shows the results of the parametric models applied to the test set compounds, and we can see that the models were reproductive and satisfactory, with residue values varying in the tetra-parametric model from ±0.67896 to ±0.02895, penta-parametric from ±0.75251 to ±0.05867, and hexa-parametric from ±0.78146 to 0.08104, see Table 5. BDBM50079321, BDBM50078426, BDBM50079322, BDBM21220 (5’-N-ethylcarboxamidoadenosine - NECA), and BDBM50385958 were the compounds that showed better prediction values with less residues.

### 2.8. Pharmacokinetic and Toxicological Predictions for the Compounds Obtained by Pharmacophore-Based Virtual Screening Approaches

In silico prediction of absorption, distribution, metabolism, excretion, and toxicity (ADMET) properties were fundamental for a quick selection of the most promising molecules for further development [20]. At this step, the 100 best-ranked compounds of each database used here (ChemBrigde_DIVERSet, ChemBrigde_DIVERSet_Exp, ZINC_Drug Database, ZINC_Natural_Stock, and ZINC_FDA_BindingD) were selected. They followed the steps of pharmacokinetic predictions (#star, “Rule of Five”, human intestinal absorption, QPPCaco, QPPMDCK, QPlogPo/w, Central Nervous System (CNS), and QPlogBB) and toxicology (waring forecast toxicity by toxicophorics groups), using the QikProp [40] and Derek softwares [40], respectively.

At the end of the process, six novel promising and potential A_2A_AR agonists were obtained: one compound of the Drug Database ZINC code ZINC00000416/MolPort-003-666-813, one of the Chembridge Diverset CL compound code 10002403, three compounds of the Chembridge Diverset EXP codes 5193875, 6942649, 7928320, respectively, as well as one compound of the ZINC Natural Code ZINC04257548/MolPort-002-509-467 (Table 6).

The pharmacokinetic and toxicological profiles resultants for the six compounds selected are shown in Table 7 and Table 8, respectively. The reference compound was also added to this pharmacokinetic and toxicological study to be a comparable source with our compounds, selected by virtual screening approaches.

By analyzing the descriptor #star and the Rule of Five (ROF) [41], the compounds obtained by virtual screening (ZINC00000416, 10002403, 5193875, 6942649, 7928320, and ZINC04257548) were found to have a common pharmacokinetic profile, with the exception of the reference compound UK-432097, which had #star = 8 and ROF = 3. The descriptor #star compares the result obtained for a compound with the observed for drugs present in the internal database of the software. The descriptor is labeled when a result is outside the 95.00% range of drug-like values. Therefore, our results showed that there were no violations of the descriptors analyzed (#star as well as the Lipinski’s Rule of Five—ROF), see Table 7.

Analysis of the descriptors for oral absorption showed that all compounds selected here had high absorption capacity and high value for human oral absorption in potential, and only compounds UK-432097, ZINC00000416, and ZINC04257548 obtained a result lower than 65.251% for prediction of the percentage of human oral absorption (HOA). However, it should be noted that compound 5193875 obtained a value of 100%, which is the maximum limit that the developer of the program considered to be high (>80%), see Table 7.

Another descriptor analyzed for oral absorption was the permeability in differentiated cells of the intestinal epithelium Caco-2 (QPPCaco), a measure used to predict absorption by the intestinal epithelium and “Madin-Darby Canine Kidney” (MDCK) (QPPMDCK) and used as a model for membrane permeability measurements [42]. Descriptors used for the prediction of passive transport should have values above 500 nm/s to be considered good, whereas values less than 25 nm/s are considered poor. Only compound 5193875 showed value greater than 500 nm/s for such a descriptor. Descriptor QPPMDCK showed maximum and minimum values of 15.137 and 485.909 nm/sec, respectively. Thus, all compounds were considered to have a low absorption according to this parameter, see Table 7.

The hydrophilic/lipophilic balance was calculated using the octanol/water partition coefficient (logPo/w), which is also an important factor for the development and optimization of compounds with pharmacological interest. The lipophilic characteristics of drugs influence their bioavailability and permeability. Lipinski [41] reported on the “Rule of Five” drug logPo/w < 5 values, according their studies with the descriptor QPlogPo/w. Here, the values calculated for all compounds selected by virtual screening varied from 0.801 < logPo/w > 4.935. Therefore, these compounds showed values within the interval accepted for drugs, see Table 7.

The ability to cross the blood–brain barrier is another important characteristic for some classes of drugs, in special compounds that have direct action in the central nervous system. However, for other therapeutical classes this property may be not recommended, since penetration of the Central Nervous System (CNS) can trigger side effects [43]. Evaluation of the selected compounds relative to the CNS descriptor revealed low penetrability ability, see Table 7.

Table 8 shows the results of toxicological predictions for UK-432097 and the other compounds selected here by virtual screening, using the DEREK software. For each molecular functional group, a “Lhasa prediction” is reported. At the end, a custom prediction (an evaluation of the molecule as a whole) is also reported. This analysis provides a criterion for excluding potentially harmful compounds in the selection step for future compound synthesis and experimental trials [44].

The structure-toxicity relationship for compound UK-432097 indicated that a toxicophoric alert for carcinogenicity was generated due to the presence of substituted pyrimidine or purine moiety in its structure, with the exception of the other compounds, which showed a toxicity prediction alert for the sensitization of skin and peroxisome proliferation as improbable (see Table 8). Pyrimidine derivatives showed carcinogenic potential, including uracil and thymine, which induce bladder carcinogenesis in rats and/or mice through formation of calculations [45]. 

The presence of a structural alert on skin sensitization within a molecule indicates that the molecule has the potential to cause skin sensitization. Regardless of the response, the molecule will be a skin sensitizer and will depend on its percutaneous absorption. Generally, small lipophilic molecules are more easily absorbed by the skin and are more susceptible to sensitization.

The presence of the toxicophoric groups within these compounds is not a guarantee that the toxic effects described will be demonstrated, but they are alerts that must be investigated experimentally in order to determine the importance of the toxicophoric groups for the development of toxic mechanisms, for future chemical modifications capable of optimizing the pharmaceutical profile of such molecules.

All compounds had a plausible toxicity alert, with the exception of compound 7928320, which besides having the warning “PLAUSIBLE” had the “IMPROBABLE” one, meaning that the probability of occurrence of toxicity is low. These results justify the fact that they went through the virtual screening, being the best among several compounds.

### 2.9. Application of QSAR Model for Compounds Selected by Virtual Screening Approaches

After the selection of six compounds by pharmacophore-based virtual screening, QSAR modeling was applied to predict in silico biological activity values. These compounds were optimized as previously described, with the descriptors of interest being extracted for the application from several models (see Table 9). The application of the QSAR models was satisfactory for compounds resulting from the virtual screening performed, especially for compounds with prediction values greater than pEC_50_>5.64936 (cutoff value based on compound **21** used in the training set).

### 2.10. Molecular Docking Studies

**Figure 6:**
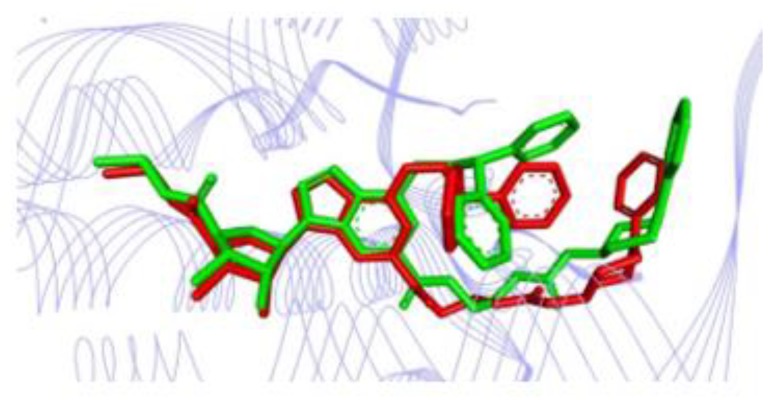
Superposition of the crystallography pose of the ligand (green) with the correspondent theoretical pose (obtained by molecular docking) for the top-ranked compound (red).

The six compounds selected here by virtual screening approaches followed the study of molecular docking. Comparison between the crystallographic ligand UK-432097 and the pose (conformation + orientation) predicted by molecular docking for our top-ranked compound can be visualized in Figure 6 and Table 10, which show the poses superimposed with RMSD of 1.58 Å. According to literature, the binding mode prediction using docking should present root mean square deviation (RMSD) value <2.0 Å when superimposed to the crystallographic pose of the ligand [46,47,48]. So, the search parameters were suitable for the docking step and the accuracy of the docking study was corroborated/validated by the crystallographic pose of the agonist into the A_2A_AR active site, reinforcing the reliability of our computational protocol to search for new potential leads or hits [23,30].

**Figure 7:**
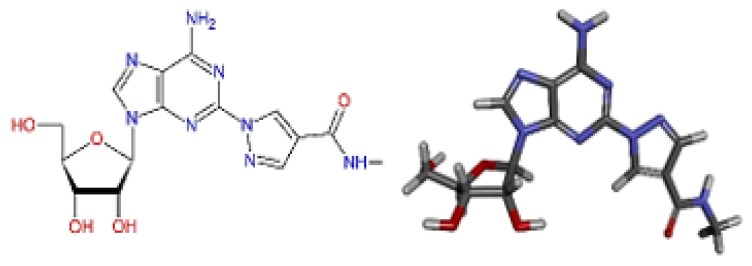
Structure of Regadenoson (CVT3146), developed by Astellas Pharma and commercially known as Lexiscan.

In the human A_2A_AR active site (PDB ID 3QAK), the compounds UK-432097 and Regadenoson (Lexiscan) were used as controls in the study of molecular docking. Despite the interaction profile of UK-432097, this compound is no more available since the Phase II clinical trials [13,17,18]. According to literature, a number of pharmacologically important agonists exhibiting A_2A_AR selectivity have been synthesized so far, such as Regadenoson, marketed as Lexiscan, an agonist approved by the Food and Drug Administration (FDA) in 2008 and marketed by Astellas Pharma. The Regadenoson agonist is a coronary vasodilator that has been found in diagnostic as well as radionuclide imaging for myocardial perfusion imaging (refers to the techniques and processes used to create images of the human body for clinical purposes) of myocardial stress acting on A_2A_AR of coronary blood vessels, which leads to dilation and a drop in blood pressure [18]. The FDA has warned of rare but serious risk of heart attack and death with cardiac nuclear stress test drugs, among them Lexiscan (regadenoson) [50]—see Figure 7.

**Figure 8:**
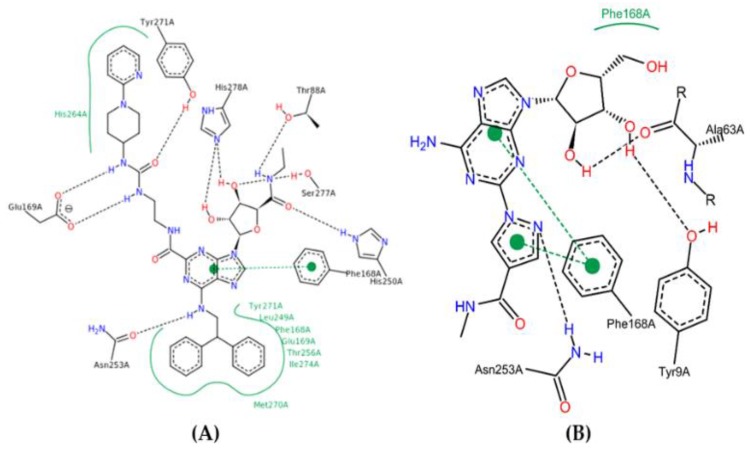
Binding profile of UK-432097 (**A**) and Regadenoson compound (**B**) with A_2A_AR. Figure generated by the Poseview webserver.

In this way, the Regadenoson compound (available as an A_2A_AR agonist) was used this study and its interaction map was generated. Using the above-mentioned docking methodology, we identified essential interactions in the A_2A_AR binding site with Regadenoson, as observed in the crystallographic pose of UK-432097 (PDB ID 3QAK), around the alpha helix located between the residues PHE168, ILE274, TYR271, and LEU249. According to the docking results obtained here, the amide group (nitrogen atoms) of the crystallography ligand UK-432097 makes a hydrogen bond with the carboxyl group of GLU169 of the receptor, while the oxygen (a hydrogen bond acceptor) makes a hydrogen bond with the phenol group of TYR271. The hydroxyls of the aryl group (a hydrogen bond donor) of UK-432097 make a hydrogen bond with the pyrimidine group of HIS278 and SER277, while the amide group near the aryl group makes a hydrogen bond with THR88 (acceptor) and HIS250 (donor). The purine nucleous of UK-432097 makes a π-stacking interaction with the phenyl group of PHE168, while TYR271, LEU249, PHE168, GLU169, THR256, ILE274, and MET270 make hydrophobic interactions with UK-432097—see Figure 8.

The phenol group of TYR9 (a hydrogen bond acceptor), the ketonic group of ALA63, and the amide group of ASN253 make a hydrogen bond with Regadenoson, while the phenol group of PHE168 makes π-stacking and hydrophobic interactions with the referred compound, as seen in others interaction maps. Thus, the π-stacking interaction with PHE168 is an essential interaction in A_2A_AR agonism—see Figure 8.

**Figure 9:**
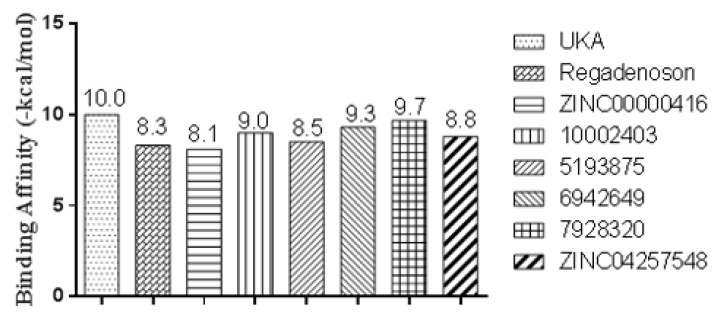
Theoretical binding affinity for UK-432097, Regadenoson, ZINC00000416, Diverset CL 10002403, Diverset EXP 5193875, Diverset EXP 6942649, Diverset EXP 7928320, and ZINC04257548 with the human adenosine A_2A_AR receptor, Protein Data Bank (PDB ID 3QAK).

Although previous studies paved the way to understanding and precise prediction using ligand-based approaches, they did not provide a molecular insight towards binding affinity and interaction mode as well. In order to accomplish this goal, results of molecular docking are shown in Figure 9 to predict the binding affinity for UK-432097, Regadenoson, and the compounds coded ZINC00000416, Diverset CL 10002403, Diverset EXP 5193875, Diverset EXP 6942649, Diverset EXP 7928320, and ZINC04257548 at the active site of A_2A_AR, which were determined at the best-ranked positions and have the most negative binding affinity, indicating a stronger binding based on the values of binding affinity. Consequently, the interactions between the receptor and the active site ligands are most significant [39].

**Figure 10:**
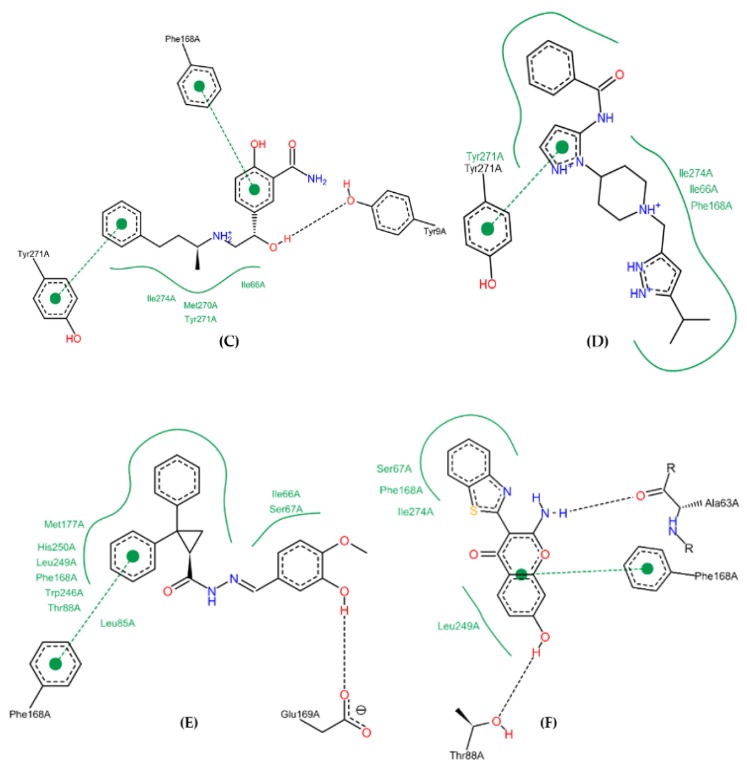
Binding profile for ZINC00000416 (**C**), Diverset CL 10002403 (**D**), Diverset EXP 5193875 (**E**) and Diverset EXP 6942649 (**F**) with the human adenosine A_2A_AR receptor, Protein Data Bank (PDB ID 3QAK). Figure generated using the Poseview webserver.

UK-432097 and Regadenoson showed binding affinity value of −10.0 kcal/mol and −8.3 kcal/mol, respectively. These values show the similarity obtained in the amino acid sequence, in which the compound Diverset EXP 7928320 had a better binding affinity value compared to Regadenoson (−9.7 kcal/mol), with a variation of ±1.4 kcal/mol, followed by Diverset EXP 6942649, Diverset CL 10002403, ZINC04257548, and Diverset EXP 5193875, with values of −9.3, −9.0, −8.8, and −8.5 kcal/mol, respectively. In comparison with ZINC00000416 (**C**) (binding energy = −8.1 kcal/mol), the compound made a hydrogen bond with TYR9 (acceptor) as well as π-stacking with PHE168 and TYR271. ILE274, MET270, TYR271, and ILE66 made hydrophobic interactions with the referred compound. Diverset CL 10002403 (**D**) (binding energy = −9.0 kcal/mol) made a π-stacking interaction with TYR271. ILE274, ILE66, and PHE168 made hydrophobic interactions with the compound (Figure 10). 

Diverset EXP 5193875 (**E**) (binding energy = -8.5 kcal/mol) compound makes a Hydrogen bond with GLU169 (a Hydrogen bond acceptor), and a π-stacking with PHE168. MET177, HIS250, LEU249, PHE168, TRP246, THR88, ILE66 and SER67 residues make hydrophobic interactions with the referred compound. The amino group of Diverset EXP 6942649 (**F**) (binding energy = -9.3 kcal/mol) makes a Hydrogen bond with ALA63 (a Hydrogen bond acceptor), while the phenol group makes Hydrogen bond with THR88. The aromatic system of Diverset EXP 6942649 makes a π-stacking interaction with the phenyl group of PHE168, while SER67, PHE168, ILE274 and LEU249 make hydrophobic interactions with the same compound (Figure 10).

**Figure 11:**
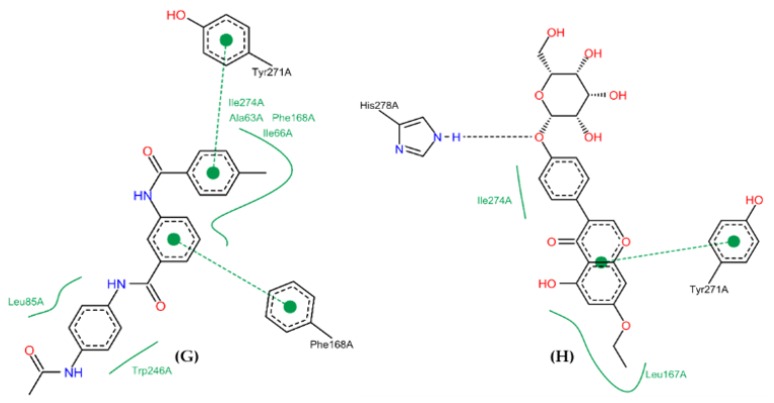
Binding profile for Diverset EXP 7928320 (**G**) and ZINC04257548 (**H**) with the human adenosine A_2A_AR receptor, Protein Data Bank (PDB ID 3QAK). Figure generated using the Poseview webserver.

In comparison with Diverset EXP 7928320 (G) (binding energy = −9.7 kcal/mol), the phenyl groups made π-stacking interactions with TYR271 and PHE168, while ILE274, ALA63, PHE168, ILE66, LEU85, and TRP246 made hydrophobic interactions. ZINC04257548 (H) (binding energy = −8.8 kcal/mol) made a hydrogen bond with HIS278 (donor) and π-stacking with TYR271. ILE274 and LEU169 residues made hydrophobic interactions with the compound (Figure 11).

Analysis of the binding mode of the crystallographic ligand inside the A_2A_AR binding site revealed that residues TYR271, LEU249, PHE168, ALA63, and ILE274 interact with the compounds investigated here, especially for PHE168 and ILE274, in particular by hydrogen bond and π-stacking interaction with the phenyl group of PHE168, whereas PHE168, ILE274, and LEU249 made hydrophobic interactions.

Although docking results are shown in our study, the interaction maps of each compound (Figure 10 and Figure 11) did not reproduce all features of the pharmacophore model built (Figure 4). When the molecules employed in the docking study are compared with pharmacophore it is possible visualize partial of stereo-electronic requirements in 2D visualization. However, this comparison cannot be performed in 3D visualization (poses obtained by molecular docking), because the molecules arrangement in active site may benefit or penalize intermolecular bonds with the chemical groups that represent each stereo-electronic requirement in pharmacophore. 

Thus, the total or partial non-visualization of stereo-electronic requirements in groups that perform map interactions may occur by the molecule adjustment in the active site, or by the selection of molecules with partial requirements in the pharmacophore step. Analysis of the of binding affinities calculated here indicated that Diverset EXP 7928320 showed good results when compared to UK-432097, being the most promising compound for A_2A_AR agonism, in terms of potential. On the other hand, the other compounds showed better binding affinity values than Regadenoson, with the exception of ZINC00000416.

A_2A_AR receptor agonists as potential agents for the treatment of rheumatoid arthritis have also been proposed but no clinical reports are yet available and research in medicinal chemistry has focused on improving the pharmacokinetic and A_2A_ selectivity profiles [50].

### 2.11. Conformational Stability of the A_2A_ Receptor-Ligand Complexes and the Binding Affinities

**Figure 12:**
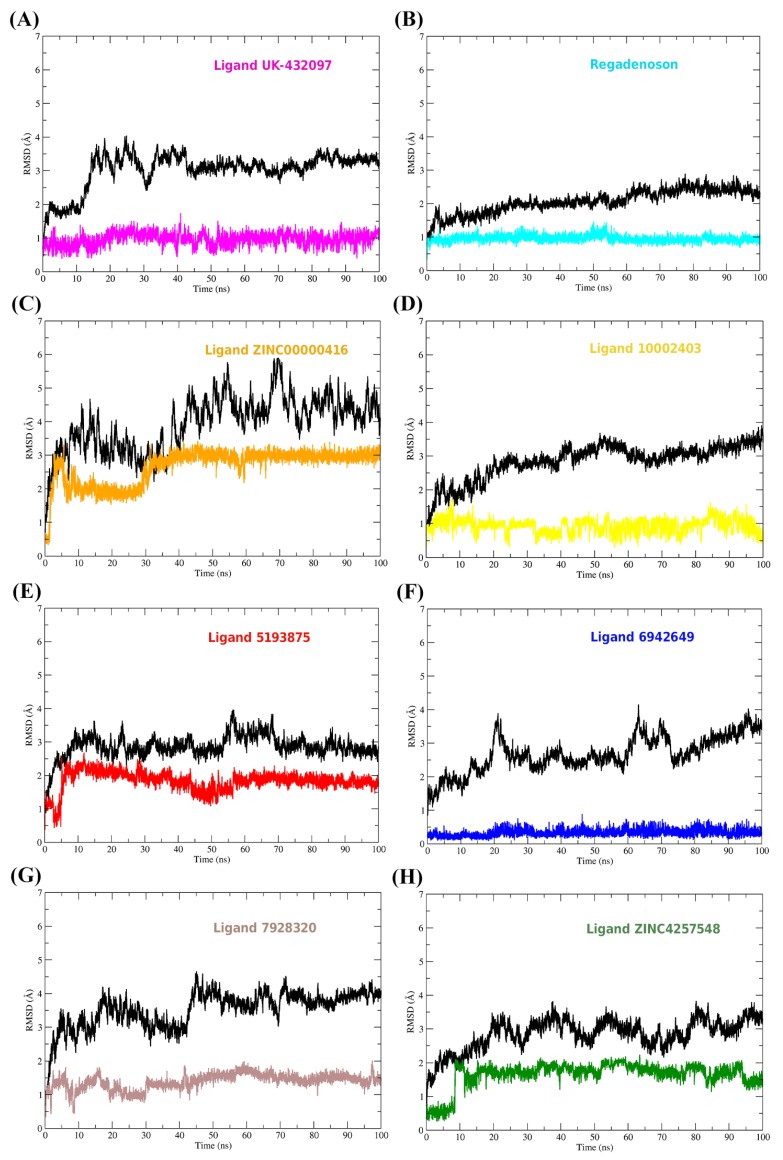
RMSD plot along the path of MD simulations. The protein backbone plot is colored black, but the ligand plots are colored in different ways. (**A**) RMSDs of the A_2A_AR-UK- 432097 system, (**B**) RMSDs of the A_2A_AR-Regadenoson system, (**C**) RMSDs of the A_2A_AR-ZINC00000416 system, (**D**) RMSDs of the A_2A_AR-Ligand 10002403 system, (**E**) RMSDs of the A_2A_AR-Ligand 5193875 system, (**F**) RMSDs of the A_2A_AR-Ligand 6942649 system, (**G**) RMSDs of the A_2A_AR-Ligand 7928320 system and (**H**) RMSDs of the A_2A_AR-ZINC4257548 system.

To evaluate conformational changes in the adenosine A_2A_ receptor and ligand structures, the Root Mean Square Deviation (RMSD) plot was plotted. To plot the RMSD of ligands all heavy atoms of the molecule were used, while for the protein RMSD plot Cα atoms were used (see Figure 12).

Throughout Molecular Dynamics (MD)simulations the ligands continued to interact with the receiver’s binding pocket. In the course of the trajectory the ligands presented different profiles in their RMSD graphs; in general the ligands showed a conformational stability tendency from about 60 ns. From this simulation time the molecules did not show drastic deviations in their conformations, thus, remained in a favorable conformation to activate the biological receptor. All ligands had negative values for affinity energy (ΔG**_bind_**), which demonstrates that the formation of receptor-ligand complexes was favorable. The affinity energy values and their energy components are shown in Table 11.

**Figure 13:**
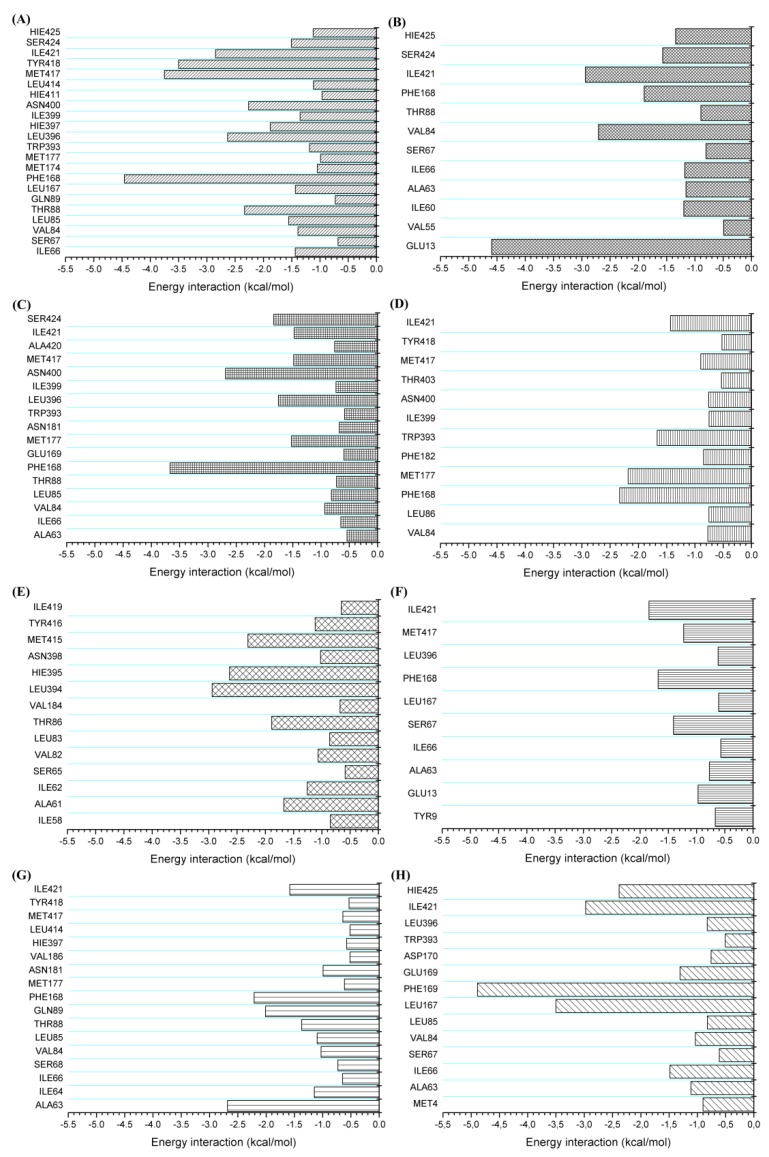
Per-residue free energy decomposition of complexes established between A_2A_ receptor and ligands (**A**) UK- 432097, (**B**) Regadenoson, (**C**) ZINC00000416, (**D**) 10002403, (**E**) 5193875, (**F**) 6942649, (**G**) 7928320 and (**H**) ZINC4257548 system.

The main energetic contributions to the A_2A_ receptor interaction with ligands were due to van der Waals contributions. To a lesser extent, electrostatic and nonpolar contributions also favored the formation of the system. Molecular interactions along molecular dynamics simulations were investigated. For this, CPPTRAJ was used to obtain the hydrogen bonds established between the receptor and ligand and also MMPBSA.py to obtain per-residue free energy decomposition. Using per-residue free energy decomposition, it was possible to quantify the interaction energy value in the complexes, as can be seen in Figure 13.

In Figure 13, it is possible to observe that the molecules were able to perform a large number of intermolecular interactions with the A_2A_AR binding site. The greatest number of interactions was observed for the crystallographic complex; however, the other compounds tested in silico also showed intense interactions with important residues of the binding cavity. Hydrogen bonds were assessed based on their occupancy time. Occupancy was defined as the percentage of time that hydrogen bonding existed during the 100 ns simulation time.

Their average distances, in angstrom (Å), were also checked. The hydrogen bonds (HBonds) were determined by the donor–acceptor atom distances ≤3.5 Å and acceptor–hydrogen donor angle of ≥ 120°. UK-432097 established strong hydrogen bonds with the residues THR88 (% = 72 and 3.1 Å; GLU169 (% = 79, 3.1 Å), HIS397 (% = 98, 2.9Å), TYR418 (% = 98, 2.7Å), HIS425 (% = 84, 2.9 Å), and ASN400 (% = 98, 2.9 Å). Molecule 5193875 interacted through HBonds with GLU169 (% = 21, 2.7 Å), LEU396 (% = 24, 3.0 Å), and ASN400 (% = 32, 2.9Å). 

The HBonds of Regadenoson were TYR9 (% = 11, 3.2 Å), GLU13 (% = 97, 2.6 Å), GLU169 (% = 29, 2.8 Å), and HIS397 (% = 47, 3 Å). The 6942649 molecule formed HBonds with three residues, GLU13 (% = 43, 2.8Å), ALA63 (% = 10, 2.9Å), and SER67 (% = 11, 3.1Å). ZINC00000416 formed HBonds with ASN181 (% = 20, 3.1Å), ASN400 (% = 25, 3.1Å), and SER424 (% = 33, 2.9Å). 

The 7928320 molecule formed HBonds with SER67 (% = 16, 2.9 Å), ASN181 (% = 87, 2.9Å), and HIS425 (% = 11, 3.0 Å). The 10002403 molecule interacted through HBonds with the residues HIS425 (% = 59, 2.8 Å) and ASN400 (% = 75, 2.9Å). ZINC04257548 formed HBonds with PHE168 (% = 31, 2.9Å), GLU169 (% = 77, 3.0 Å), ASP170 (% = 26, 2.7 Å), and HIS425 (% = 98, 2.9 Å).

## 3. Materials and Methods 

### 3.1. Pharmacophore Detection by Pharmagist and Discovery Studio 4.0 

**Figure 14:**
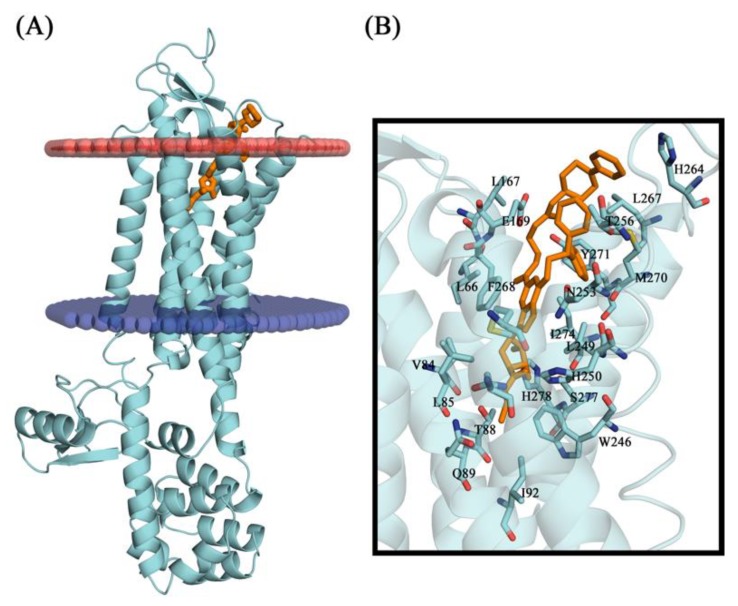
(**A**) Schematic representation of the type 2 adenosine receptor. In red the extracellular side is represented and blue represents the cytoplasmic side. (**B**) Binding site of the protein with the compound UK-432097.

Initially, the A_2A_AR crystallographic (PDB ID: 3QAK - at 2.71 Å resolution) was obtained as a PDB file from the Protein Data Bank (http://www.rcsb.org/pdb/home/home.do) in complex with 6-(2,2-diphenylethylamino)-9-[(2R,3R,4S,5S)-5-(ethylcarbamoyl)-3,4-dihydroxy-oxolan-2-yl]-*N*-[2-[(1-pyridin-2-ylpiperidin-4-yl)carbamoylamin]purine-2-carboxamide (UK-432097) [18], see Figure 14.

**Figure 15:**
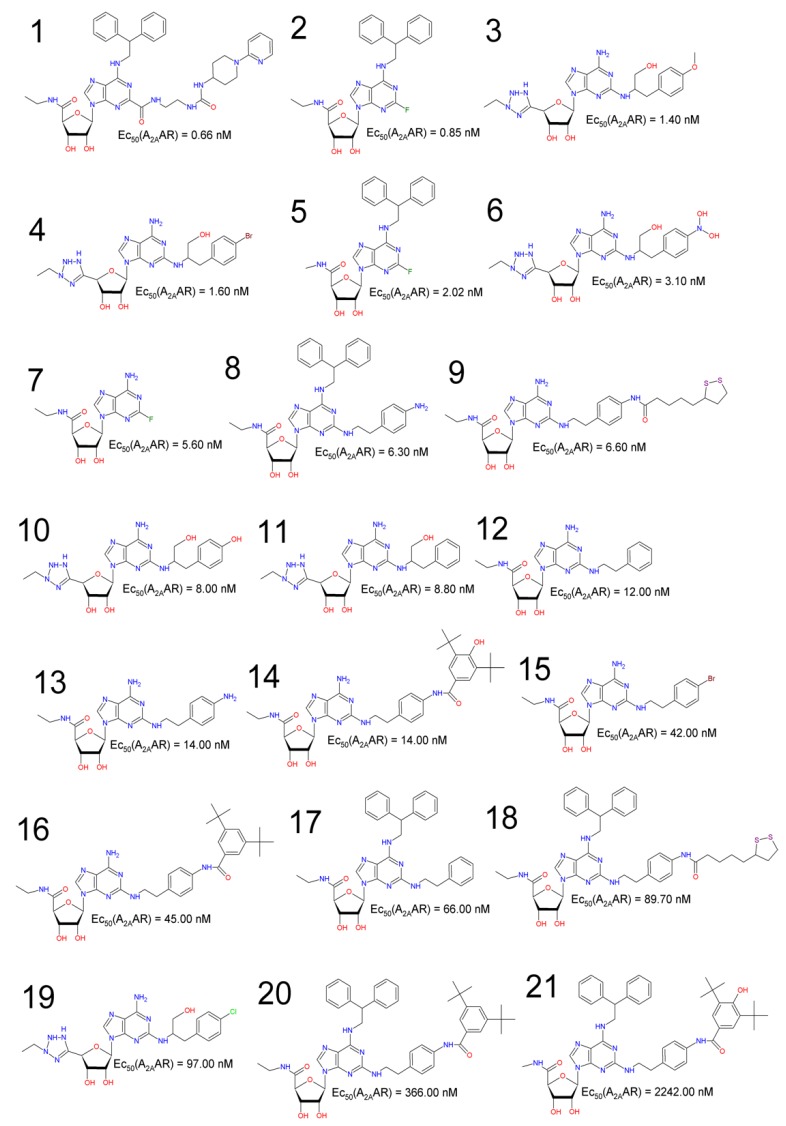
Selected compounds with their respective EC_50_ (A_2A_AR) values.

The selection of the A_2A_AR agonists, was done in the literature and Pubchem database (https://pubchem.ncbi.nlm.nih.gov) according to their EC_50_ values—see Table 12 and Figure 15. The selection criteria used here included compounds with EC_50_ between 0.66 and 2242.00 nM, which are compounds derived from adenosine and presenting an adenine moiety as well as a tetrahydrofuran-3,4-diol moiety (a ribose derivative). 

These compounds were pre-aligned using pharmacophore perception with PharmaGist Web Server (http://bioinfo3d.cs.tau.ac.il/pharma/index.html) and refined using the Discovery Studio software. Compounds analyzed were obtained from cAMP assays on CHO cells expressing the adenosine receptor type 2A (A_2A_AR), according to studies developed by Hausler et al. [16], Xu et al. [18] and Kim et al [51].

### 3.2. Pharmacophore Prediction 

Pharmacophore hypothesis was given by the alignment of compounds, identifying the common shared regions. The identification of a pharmacophore can serve as an important model in the rational drug design, since it can aid in the discovery of novel compounds that could bind to a target receptor and pharmacophore prediction provides the spatial arrangement of molecular features that are essential for target-ligand interactions [52,53]. The common pharmacophore previously established was examined using a scoring function to achieve the best alignment for the 21 most active compounds investigated here.

The PharmaGist web server [34] and the Discovery Studio 4.0 software were used to generate the pharmacophore model using the HipHop method. The pharmacophore model chosen in pre-alignment with a better score was used as input in the Discovery Studio, in order to refine the original pharmacophore model to start the virtual screening process. The advantage of HipHop method stems from the use of the three-dimensional conformation of the crystallographic structure of the ligand-protein complex. Thus, during the analysis of the pharmacophore hypotheses we excluded the assumptions that were different from the expected interactions reported for the active site of the A_2A_AR receptor. This was based on the common characteristics present in the active molecules chosen to derive the refereed pharmacophore model, using the UK-432097 agonist (PDB ID 3QAK—the crystallographic pose) as spatial template/pivot to be superimposed to the ligands [54].

Pharmacophore model evaluation/validation was performed through a “contaminated” base of one part of the subcollecion containing 30 reference compounds (15 agonists and 15 antagonists). The database used for the pharmacophore model contained 500 random unknown compounds from the Diversety database. The most effective model was used in pharmacophore-based virtual screening procedures, in subsequent steps using the Discovery Studio 4.0 software.

### 3.3. Correlation Analysis, Design and Validation of the Multiple Linear Regression Model

The most active compounds had their geometry pre-optimized using molecular mechanics, to minimize energy and subsequently using the semi-empirical Hamiltonian method PM3 [55] with a gradient norm threshold of 0.01 kcal/A.mol, using the HyperChem software 8.0.

For the construction of the multiple linear regression model, 18 descriptors were obtained. Pharmacophore descriptors (Pharmacophore Features—PF) were obtained through the PharmaGist web server [35], such as, hydrogen bond donor (HBD), hydrogen bond acceptor (HBA), anion. After obtaining all descriptors, one data matrix was built, and before starting the analysis it was necessary to do the standardization, i.e., the autoescale of the data matrix X = (n,m) containing 21 lines (compounds studied) and 18 columns (descriptors), where n is the number of compounds studied and m is the number of variables.

The objective of autoescale was to give each descriptor equal weight in mathematical terms and each variable was centered on the average, being scaled to unit variance. As the biological data of EC_50_ (nM) were obtained from different sources, the EC_50_ values were converted to pEC_50_ = −logEC_50_, in order to reduce the inconsistencies caused by experimental steps [30].

Posteriorly, the Pearson correlation was used to identify the descriptors among the 21 most active compounds associated with the A_2A_AR activity. In this work, the correlation cutoff used was 0.5, according to studies carried out by Pereira [52] and Santos [56].

In order to test the predictive power of the QSAR model, their validation was made by calculating the error of the compounds to build the models as well as regarding the statistical data. After identifying the main properties associated with the biological activity, a penta-parametric model multiple linear regression model (MLR) was designed.

Pearson correlation study and the multiple linear regression modeling were performed using the Statistica 7 (v. 7.0.61.0) software (available in http://www.statsoft.com/) to estimate biological activity values of selected compounds by virtual screening procedures, the multiple linear regression model was applied for the compounds screened that did not have activity values (EC_50_) reported.

### 3.4. Pharmacophore-Based Virtual Screening

The virtual screening was performed with the databases ChemBrigde_DIVERSet, ChemBrigde_DIVERSet_Exp, ZINC_Drug Database, ZINC_Natural_Stock, and ZINC_FDA_BindingD in the Discovery Studio 4.0 program, using the best pharmacophore model. Based on the study of Mantoani [57], initially, for each molecule in the database, the fast conformer generation method was used with a maximum energy tolerance of 20 kcal/mol. In the Discovery Studio 4.0 software [35], two database search options were then employed: “Fast/Flexible” and “Best/Flexible”. 

The first filtration was performed in order to obtain the “Top 5000” compounds (according to the FitValue) for each base (“Fast” generation of conformers). In sequence, the compounds with better fit to the pharmacophore models were selected. Subsequently, a second virtual screening was performed using the “Best/Flexible” option in order to obtain the “Top 100” (according to the FitValue) of each base, selecting 500 compounds that followed for the subsequent stage of pharmacokinetic and toxicological predictions.

### 3.5. Pharmacokinetic and Toxicological Predictions 

Pharmacokinetic and toxicological studies were performed for compounds resultant of the virtual screening step (Top 100) of each base, with better values based on the pharmacophore model. Pharmacokinetic (#star, “Rule of Five”, Human intestinal absorption, QPPCaco, QPPMDCK, QPlogPo/w, CNS and QPlogBB) and toxicological (waring forecast toxicity by toxicophorics groups) properties were predicted using the Shrodinger’s Suite QikProp [40] and Derek Nexus Softwares [44].

### 3.6. Molecular Docking Studies

Compounds and A_2A_AR receptor structure were prepared using the Discovery Studio 4.0 software. A_2A_AR receptor in complex with UK-432097 (PDB ID 3QAK—the crystallographic pose) [18] and compounds with satisfactory results in previous steps (virtual screening) were used for molecular docking studies with the AutoDock Vina 1.1.2 software [58] and the graphical interface PyRx version 0.8.30 (available in https://pyrx.sourceforge.io) [59], respectively. 

Root Mean Square Deviation (RMSD) value was calculated in order to validate the molecular docking results, by comparison of crystallographic ligand (UK-432097) and the best theoretical pose obtained. First, an initial docking box was constructed to enclose the bound ligand, and then the box size was increased until it included the entire region of the active site to allow rotation and translation of the ligand inside this region.

Molecular docking was performed to obtain a population of possible conformations and orientations for the ligand at the receptor binding site. The protein was loaded in PyRx, creating a PDBQT file that contained a protein structure with hydrogens in all polar residues. All calculations for protein fixed ligand-flexible docking were done using the Lamarckian Genetic Algorithm (LGA) method, which presented the best results in the search for the global minimum [60]. 

The docking site on the protein target was defined by establishing a grid box with the dimensions shown Table 13. Ten runs via AutoDock Vina, with exhaustiveness default = 8, were performed in all cases for each ligand structure, and for each run the best pose with the lowest binding free energy and lower RMSD was selected, saved, and analyzed. 

RMSD was calculated using the Discovery Studio Visualizer software, by comparing the crystallographic ligand pose and the docking pose, using all atoms. The X, Y, and Z spatial coordinates were determined in the active site region according to the observed interaction between the receptor and their respective crystallographic ligand. The coordinates used for the center and the box size can be seen in Table 13. 

The interaction maps visualization between compounds and A_2A_AR was performed using the Poseview web server (available in https://www.biosolveit.de/PoseView/). In this work, the compounds UK-432097 and Regadenoson^®^ (Lexiscan) were used as positive controls.

### 3.7. Molecular Dynamics Simulations

The initial poses for each complex were obtained from the molecular docking results, as described in the previous section. The restrained electrostatic potential (RESP) protocol with the HF/6-31G* [61] basis sets was applied to obtain the atomic charges of the atoms of each ligand, the charge calculated using Gaussian 16 software [62]. The parameters of the ligand were constructed with the Antechamber module, being described by General Amber Force Field (GAFF) [63]. 

The protonation state of ionizable residues of receptor structure was analyzed using the PROPKA server [64] in the neutral pH. Receptor-ligand systems were embedded in a pre-equilibrated 1-palmitoyl-2-oleoyl-sn-glycero-3-phosphoethanolamine (POPE) bilayer membrane. The embedding of the system into the POPE membrane was performed using the PPM server (available in https://opm.phar.umich.edu/ppm_server) [65] and CHARMM-GUI [66]. Protein parameters were described by force field 14SB [67], whereas lipids were described by Lipid14 [68]. The water molecules used in MD simulations were TIP3P [69]. An appropriate number of counterions were added to neutralize the total charge of the complexes.

The sander.MPI and pmemd.CUDA of the Amber 16 package [70,71] was used to perform molecular dynamics (MD) simulations. System energy was minimized using a combination of steepest descent and conjugate gradient methods. A total of 1000 cycles of steepest descent and 2000 cycles of the conjugate gradient were used to minimize the energy of molecules and water and counter ions that made up the system. Then, the position of the hydrogen atoms of the protein and ligands were minimized with 3000 steps of the steepest descent algorithm and 2000 steps of the conjugate gradient. 

The systems were heated from 0 to 300 K in two steps, first 2 ns of MD simulations with NVT ensemble were used to raise the system temperature from 0 to 100 K. In the second step 5 ns of MD simulations were generated to raise the temperature up to 300 K with the NPT ensemble with 1 atm pressure using the Berendsen barostat. After all these steps, 100 ns production runs were performed for each complex. The particle mesh Ewald method [72] was used for the calculation of the electrostatic interactions, and the bonds involving hydrogen atoms were restricted with the SHAKE algorithm [73]. The temperature control was performed with a Langevin [74] thermostat within a collision frequency of 2 ps^−1^.

### 3.8. Binding Affinities Calculations

To estimate the A_2A_ receptor-ligand, affinity energy was calculated with the Molecular Mechanics/Generalized Born Surface Area (MM/GBSA) method [75,76]. For our calculations we used 500 snapshots of the last 5 ns of MD simulation.

The free energy was estimated according to Equation (6):

ΔG_bind_ = ΔE_MM_ + ΔG_solv_ – TΔS, (6)

where ΔG_bind_ is the affinity energy resulting from the sum of the total energy in the gas phase (ΔE_MM_), free energy of solvation (ΔG_solv_), and entropy (TΔS).

ΔE_MM_ is the sum of ΔE_internal_ (connections, angles, and dihedra), ΔE_electrostatic_ (electrostatic contributions), and ΔE_vdW_ (Van der Waals contributions), according to Equation (7):

ΔE_MM_ = ΔE_internal_ + ΔE_electrostatic_ + ΔE_vdw_. (7)

ΔG_solv_ can be obtained from the resolution of Equation (8):

ΔG_solv_ = ΔG_GB_ + ΔG_SASA_, (8)

where the polar contributions (ΔGGB) are calculated using the GB model and the non-polar contributions (ΔGSASA) are determined from the calculation of the solvent accessible surface area (SASA).

### 3.9. Per-Residue Free Energy Decomposition Analysis

Per-residue free energy decomposition was decomposed using the approach of MM/GBSA according to the equation [77,78,79,80,81]:

ΔG_MM-GBSA_ = ΔE_vdW_ + ΔE_elec_ + ΔE_pol_ + ΔE_np_. (9)

## 4. Conclusions

After virtual screening in the commercial databases, six new promising compounds for anti-inflammatory activity were obtained to bind the adenosine A_2A_AR, and a search on SciFinder^®^, available online in the Chemical Abstract Service (CAS) (https://scifinder.cas.org/), was realized to obtain additional information on structures with biological activities and no further information on the compounds selected in the search was found, only information on some physicochemical properties already reported in the database, and after a search in several databases, no study was found on a possible biological activity for which this research was proposed. 

The vast literature and numerous research tools of medicinal chemistry available make the A_2A_AR a target of great interest, but not only about receptor distribution, but study into its structure as from X-ray crystal structures serves to the design of novel potent and selective A_2A_ ligands. Development of a multiple linear regression model to predict the pEC_50_ value of virtual screening compounds was satisfactory. After application of the QSAR model, pharmacokinetic, and toxicological studies, we showed that compound 5,193,875 (Chembridge Diverset EXP with pEC_50_ = 6.06614) exhibited the best oral absorption and bioavailability in potential, while presenting low CNS values, which demonstrates low cerebral permeability. In addition, compounds selected here using virtual screening procedures showed common toxicity alerts; although they are plausible, the toxicophoric group is present in molecules in the human body metabolism. In conclusion, considering the low EC_50_ value and high pEC_50_ value, only two compounds have promising activity due to their satisfactory ADME/Tox profiles and predictions via QSAR (Diverset CL 10,002,403 with pEC_50_ = 7.54407 and ZINC04257548 with pEC_50_ = 7.38310). Moreover, satisfactory molecular docking results were obtained when compared to the Regadenoson (Lexiscan^®^), used here as a positive control. These compounds should be used in biological assays (in vitro and in vivo) in order to confirm the potential activity agonist to A_2A_AR.

## Figures and Tables

**Table 1:** Coordinates and radius of the spatial features of the selected pharmacophore model.

Spatial Characteristics	X	Y	Z	Radius
Aromatic 1 (AR1)	−5.449	−10.056	53.789	1.1
Aromatic 2 (AR2)	−4.468	−9.125	52.185	1.1
Hydrogen Acceptor 1 (HA1)	−5.990	−10.020	55.012	0.5
Hydrogen Acceptor 2 (HA2)	−4.278	−8.013	52.455	0.5
Hydrogen Acceptor 3 (HA4)	−5.467	−11.213	52.094	0.5
Hydrogen Acceptor 4 (HA4)	−5.735	−10.642	47.065	0.5

**Table 2:** Molecular descriptors selected for QSAR modeling.

Compound	CODE	HG ^a^	PF ^b^	NA ^c^	MP ^d^	MV ^e^	AR ^f^
**1**	UK-432097	3	26	104	82.39	2155.82	5
**2**	BDBM50385948	3	19	64	52.03	1379.46	4
**3**	BDBM50150762	3	25	67	51.53	1393.64	3
**4**	BDBM50150765	2	23	63	51.69	1381.93	3
**5**	BDBM50385955	3	20	61	50.20	1317.10	5
**6**	BDBM50150764	2	26	65	51.69	1420.28	4
**7**	BDBM50385957	1	16	38	29.04	846.90	3
**8**	BDBM50385950	4	23	84	68.15	1755.00	5
**9**	BDBM50385947	4	24	81	65.16	1738.51	3
**10**	BDBM50150767	2	25	64	49.70	1361.57	3
**11**	BDBM50150766	2	23	63	49.06	1339.60	3
**12**	BDBM50385958	2	19	56	43.81	1205.69	4
**13**	BDBM50385943	2	20	58	45.16	1243.14	4
**14**	BDBM50385946	16	38	95	72.06	1895.57	5
**15**	BDBM50385944	2	18	56	46.44	1271.34	3
**16**	BDBM50385945	16	36	94	71.43	1876.10	5
**17**	BDBM50385949	4	22	82	66.80	1720.20	5
**18**	BDBM50385954	6	28	107	88.15	2257.04	5
**19**	BDBM50150763	2	24	63	50.99	1371.19	4
**20**	BDBM50385952	18	39	120	94.42	2393.81	6
**21**	BDBM50385956	18	41	118	93.22	2352.49	6

^a^ Hydrophobic Group; ^b^ Pharmacophore Features; ^c^ Number of Atoms; ^d^ Molecular Polarizability; ^e^ Molar Volume (A^3^); ^f^ Aromatic.

**Table 3:** Internal validation using the best built QSAR models (tetra-, penta- and hexaparametic).

Compound	Parametric QSAR Models (pEC_50_ = −logEC_50_)						Experimental (pEC_50_)
	Tetra-	Residual Values	Penta-	Residual Values	Hexa-	Residual Values	
**1**	8.98294818	0.197512	9.12779843	0.052662	8.642608925	0.537851	9.18046
**2** *	6.99156978	2.07901	7.13790683	1.932673	6.762230924	2.308349	9.07058
**3** *	8.27890486	0.574965	8.12289461	0.730975	7.763229123	1.090641	8.85387
**4** *	7.20833204	1.587548	6.98392019	1.81196	6.623110462	2.17277	8.79588
**5** *	6.4390024	2.255648	6.3982794	2.296371	5.974691405	2.719959	8.69465
**6**	8.39284814	0.115792	8.11686529	0.391775	7.793326136	0.715314	8.50864
**7**	7.76172650	0.490083	7.8997063	0.352104	7.565734821	0.686075	8.25181
**8** *	6.92233110	1.278329	6.94590035	1.25476	6.46444254	1.736217	8.20066
**9**	8.10517950	0.075281	8.2346837	−0.054224	7.958933784	0.221526	8.18046
**10**	8.45979122	−0.362881	8.24137752	−0.144468	7.887637662	0.209272	8.09691
**11**	8.27402464	−0.218505	8.18126794	−0.125748	7.819516242	0.236004	8.05552
**12**	7.91841568	0.002404	8.05102783	−0.130208	7.691452796	0.229367	7.92082
**13**	8.11225228	−0.258382	8.20539668	−0.351527	7.844506896	0.009363	7.85387
**14**	7.56453846	0.289332	7.54459856	0.309271	7.402379418	0.451491	7.85387
**15**	7.30993500	0.066815	7.4768018	−0.100052	7.194105882	0.182644	7.37675
**16**	7.42057922	−0.073789	7.53430987	−0.18752	7.389967518	−0.043178	7.34679
**17**	6.78045040	0.40001	6.8531334	0.327327	6.37965774	0.800802	7.18046
**18**	7.04603034	0.00118	7.04921319	−0.002003	6.619084889	0.428125	7.04721
**19**	7.50229980	−0.48907	7.23382085	−0.220591	6.856821156	0.156409	7.01323
**20**	6.34535314	0.091167	6.41080264	0.025717	6.027298653	0.409221	6.43652
**21**	5.96752642	−0.318166	5.79898512	−0.149625	5.399238413	0.250122	5.64936

* Outliers; Residual Values = calculated by the difference between the experimental and the theoretical values.

**Table 4:** Molecular descriptors selected for QSAR modeling.

Compound	Code	MV ^a^	MP ^b^	NA ^c^	PF ^d^	HG ^e^	AR ^f^	EC_50_ (nM)
**22**	BDBM35804 (CGS21680)	1383.25	50.59	64	22	3	3	2.12
**23**	BDBM50079321	1487.3	54.92	70	18	1	3	4.89
**24**	BDBM50026816	1834.39	66.65	81	27	4	4	5.86
**25**	BDBM50078426	1042.47	36	45	19	2	3	9.75
**26**	BDBM50079322	1395.7	52.9	70	18	1	3	10.16
**27**	BDBM21220 (NECA)	855.09	29.13	38	15	1	2	12.58
**28**	BDBM50385958	1218.48	43.81	56	18	2	3	12.00

^a^ Molar Volume (A^3^); ^b^ Molecular Polarizability; ^c^ Number of Atoms; ^d^ Pharmacophore Features; ^e^ Hydrophobic Group; ^f^ Aromatic.

**Table 5:** External validation using the best built QSAR models (tetra-, penta-, and hexaparametic) with selected compounds from the Pubchem database.

Compound	Parametric QSAR Models						Experimental (pEC_50_) ^b^
	Tetra-	Residual Values ^a^	Penta-	Residual Values ^a^	Hexa-	Residual Values ^a^	
BDBM35804 (CGS21680)	8.10378	0.56982	8.17729	0.49631	7.89214	0.78146	8.6736
BDBM50079321	8.59992	−0.28932	8.92832	−0.61772	8.51537	−0.20477	8.3106
BDBM50026816	8.91106	−0.67896	8.98461	−0.75251	8.85516	−0.62306	8.2321
BDBM50078426	7.96806	0.04284	8.06957	−0.05867	7.85361	0.15729	8.0109
BDBM50079322	8.25996	−0.26686	8.4744	−0.4813	7.91206	0.08104	7.9931
BDBM21220 (NECA)	7.87135	0.02895	8.11297	−0.21267	7.80671	0.09359	7.9003
BDBM50385958	8.16918	−0.24838	8.42937	−0.50857	8.11924	−0.19844	7.9208

^a^ Residual Values = calculated by the difference between the experimental and the theoretical values. ^b^ pEC_50_ = −logEC_50_.

**Table 6:** Compounds selected by pharmacophore-based virtual screening of future purchase and biological assays (3QAK/UK-432097).

Compounds	Database	Code
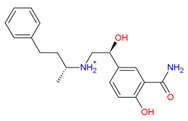	Drug Database ZINC	ZINC00000416 MolPort-003-666-813
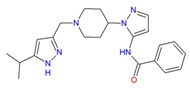	Chembridge Diverset CL	10002403.
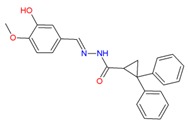	Chembridge Diverset EXP	5193875.
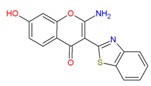	Chembridge Diverset EXP	6942649.
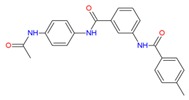	Chembridge Diverset EXP	7928320.
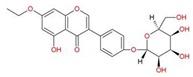	Natural ZINC	ZINC04257548 MolPort-002-509-467

**Table 7:** Molecular properties calculated for evaluation of metabolism and pharmacokinetic profiles of UK-432097 and compounds selected by virtual screening approaches.

Compound	HOA ^a^	%HOA ^b^	QPPCaco ^c^	QPPMDCK ^d^	QPlog*P*o/w ^e^	CNS ^f^	QPlogBB ^g^
UK−432097	Medium	27.582	24.065	17.281	2.526	−2	−3.029
ZINC00000416	Medium	70.681	36.174	15.137	2.706	−2	−1.582
10002403	High	92.066	293.027	145.218	3.581	1	−0.328
5193875	High	100.000	983.532	485.909	4.935	−1	−0.842
6942649	High	82.447	309.168	216.994	1.867	−1	−0.938
7928320	High	93.856	441.554	204.465	3.342	−2	−1.382
ZINC04257548	Medium	65.251	75.562	30.334	0.801	−2	−2.344

^a^ Human Oral Absorption (HOA), ^b^ Human oral absorption (%HOA), ^c^ permeability of the differentiated cells of the intestinal epithelium Caco-2 (QPPCaco), ^d^ Madin-Darby Canine Kidney (QPPMDCK), ^e^ apparent permeability of compound between octanol/water (QPlogPo/w), ^f^ Central Nervous System (CNS), ^g^ apparent permeability of compound in the blood–brain barrier (QPlogBB).

**Table 8:** Toxicity prediction for UK-432097 and compounds selected by virtual screening.

Compound Code	Toxicity Prediction Alert (Lhasa Prediction)	Toxicophoric Group	Toxicity Alert	Toxicity Prediction (Custom Prediction)
UK-432097	Carcinogenicity	Pyrimidine or substituted purine	PLAUSIBLE	Nothing to declare
ZINC00000416	Skin sensitization	Phenol or precursor	PLAUSIBLE	Nothing to declare
10002403	-	-	-	Nothing to declare
5193875	Skin sensitization	Catechol or precursor; Hydrazine or precursor	PLAUSIBLE	Nothing to declare
6942649	Skin sensitization	Phenol or precursor	PLAUSIBLE	Nothing to declare
7928320	Proliferation of peroxisome	Alkyl aryl Bi aryl carboxylic acid or precursor	IMPROBABLE	Nothing to declare
	Skin sensitization	Primary or secondary aromatic amine	PLAUSIBLE	
ZINC04257548	Skin sensitization	Enol ether	PLAUSIBLE	Nothing to declare
		Resorcinol or precursor		

**Table 9:** Compounds selected by virtual screening and descriptors used in the QSAR model.

Compound Code	Molecular Properties						Parametric QSAR Models (pEC_50_ = −logEC_50_)		
	MV ^a^	MP ^b^	NA ^c^	PF ^d^	HG ^e^	AR ^f^	Tetra-	Penta-	Hexa-
ZINC00000416	990.05	37.00	48	13	3	2	6.72890	7.16159	6.78904
10002403	1188.63	44.45	57	15	4	3	7.05978	7.54407	7.21051
5193875	1141.29	43.64	52	13	4	3	5.63389	6.06614	5.74906
6942649	802.06	32.34	32	11	0	4	3.35970	3.16129	2.66112
7928320	1144.49	43.24	50	12	3	3	5.69190	6.14174	5.84216
ZINC04257548	1183.02	44.36	57	19	1	3	7.43343	7.38310	6.89273

^a^ Molar Volume (A^3^); ^b^ Molecular Polarizability; ^c^ Number of Atoms; ^d^ Pharmacophore Features; ^e^ Hydrophobic Group; ^f^ Aromatic.

**Table 10:** Comparison between experimental and theoretical binding affinities.

Receptor	Ligand	Experimental Binding Affinity * (kcal/mol)	Ki (nM) *h*A_2A_R	Docking Predicted Binding affinity (kcal/mol)	Resolution (Å)
PDB ID 3QAK	UK-432097	−11.45	4.00 [39,49]	−10.00	2.71
		−11.35	4.75 [18]		

* Values calculated from experimentally determined inhibition constants (Ki), reported in the PDB, according to Equation: ΔG = R.T.lnKi, were R (constant of gas) = 1.987.10^−3^ kcal.mol^−1^.K^−1^ and T (temperature) = 298.15 K.

**Table 11:** Affinity energy values and energy components. ΔE_vdW_, Van der Waals contributions; ΔE_ele_, electrostatic contributions; ΔG_GB_, polar contributions; ΔG_np_, non-polar contributions; ΔG_bind_, affinity energy.

Ligand	Terms				
	ΔE_vdW_	ΔE_ele_	ΔG_GB_	ΔG_NP_	ΔG_bind_
Regadenoson	−51.85 ± 0.18	−20.22 ± 0.37	59.03 ± 0.22	−33.02 ± 0.01	−46.06 ± 0.25
UK-432097	−49.78 ± 0.23	−31.63 ± 0.44	41.03 ± 0.32	−11.23 ± 0.01	−51.61 ± 0.28
ZINC00000416	−47.88 ± 0.15	−23.78 ± 0.24	36.43 ± 0.14	−6.84 ± 0.01	−42.07 ± 0.19
10002403	−48.16 ± 0.20	−21.59 ± 0.32	35.70 ± 0.24	−6.50 ± 0.01	−40.55 ± 0.25
5193875	−48.62 ± 0.15	−7.92 ± 0.21	23.69 ± 0.15	−7.51 ± 0.01	−40.36 ± 0.18
6942649	−28.31 ± 0.15	−23.15±0.31	24.51 ± 0.39	−6.44 ± 0.01	−33.39 ± 0.22
7928320	−45.57 ± 0.18	−33.31 ± 0.43	46.36 ± 0.31	−6.44 ± 0.01	−38.96 ± 0.20
ZINC04257548	−49.83 ± 0.19	−42.54 ± 0.55	58.17 ± 0.37	−7.15 ± 0.01	−41.35 ± 0.22

**Table 12:** Compounds selected, BindingDB codes and their respective biological activity values.

Compound	Code	EC_50_ (nM)	pEC_50_ [a]	Reference
**1**	UK-432097	0.66	9.18046	[18]
**2**	BDBM50385948	0.85	9.07058	[16]
**3**	BDBM50150762	1.40	8.85387	[51]
**4**	BDBM50150765	1.60	8.79588	[51]
**5**	BDBM50385955	2.02	8.69465	[16]
**6**	BDBM50150764	3.10	8.50864	[51]
**7**	BDBM50385957	5.60	8.25181	[16]
**8**	BDBM50385950	6.30	8.20066	[16]
**9**	BDBM50385947	6.60	8.18046	[16]
**10**	BDBM50150767	8.00	8.09691	[51]
**11**	BDBM50150766	8.80	8.05552	[51]
**12**	BDBM50385958	12.00	7.92082	[16]
**13**	BDBM50385943	14.00	7.85387	[16]
**14**	BDBM50385946	14.00	7.85387	[16]
**15**	BDBM50385944	42.00	7.37675	[16]
**16**	BDBM50385945	45.00	7.34679	[16]
**17**	BDBM50385949	66.00	7.18046	[16]
**18**	BDBM50385954	89.70	7.04721	[16]
**19**	BDBM50150763	97.00	7.01323	[51]
**20**	BDBM50385952	366.00	6.43652	[16]
**21**	BDBM50385956	2242.00	5.64936	[16]

[a] pEC_50_ = -logEC_50._

**Table 13:** Protocols used for the molecular docking study.

Receptor	Ligand *	Coordinates of the Grid Center (Angstrom)	Grid dimensions (Angstrom)
A_2A_AR (PDB ID 3QAK)	UK-432097	X = −7.076 Y = −9.074 Z = 55.148	X = 52 Y = 38 Z = 60

* The agonist UK-432097 (6-(2,2-diphenylethylamino)-9-((2R,3R,4S,5S)-5-(ethylcarbamoyl)-3,4- dihydroxytetrahydrofuran-2-yl)-*N*-(2-(3-(1-(pyridin-2-yl)piperidin-4-yl)ureido)ethyl)-9H-purine-2- carboxamide) was used as control in the molecular docking validation.
